# Diversity of ejaculated sperm proteins in Moxotó bucks (*Capra hircus* ) evaluated by multiple extraction methods

**DOI:** 10.21451/1984-3143-2017-AR966

**Published:** 2018-08-16

**Authors:** Raulzito Fernandes Moreira, Maria Nágila Carneiro Matos, João Garcia Alves, Roberta Vianna do Valle, Angela Maria Xavier Eloy, Tatiana Maria Farias Pinto, Saris Pinto Machado, Cíntia Renata Rocha Costa, José Luiz de Lima, João Paulo Matos Santos Lima, Rodrigo Maranguape Silva da Cunha

**Affiliations:** 1 Departamento de Biotecnologia, Universidade Federal do Ceará (UFC), programa de pós-graduação em biotecnologia (PPGB), Sobral, CE, .; 2 Departamento de Zootecnia, Universidade Estadual Vale do Acaraú (UVA), Programa de Pós-Graduação em Zootecnia (PPGZ), Sobral, CE,; 3 Núcleo de Biotecnologia de Sobral (NUBIS), Universidade Estadual Vale do Acaraú (UVA), Sobral, Ceará, .; 4 Empresa Brasileira de Pesquisa Agropecuária (EMBRAPA Caprinos e Ovinos), Sobral, CE, .; 5 Núcleo de Biologia Experimental (Nubex), , , .; 6 Laboratório de imunopatologia keizo Asami (LIKA), Departamento de Bioquímica, , , .; 7 Departamento de Bioquímica, Universidade Federal do Rio Grande do Norte, Natal, Brasil. Endereço: Campus Universitário Lagoa Nova, , .

**Keywords:** detergents, isolation methods, proteomic profiles, spermatozoids

## Abstract

This study aimed to develop protocols for the extraction of sperm proteins from Moxotó goats (*Capra hircus*) and to compare the resulting proteomic maps. The sperm proteins were isolated using an extraction buffer containing 7 M urea and 2 M thiourea, 20 mM DTT, and one of the following detergents: 1% or 4% CHAPS; 1% or 4% SDS; 1% or 4% Triton X-100; or a combination of CHAPS and SDS. The 1-DE and 2-DE profiles of the isolated proteins revealed that the various isolation methods were efficient. Qualitative and quantitative differences in the 1-DE and 2-DE profiles were observed. 2-DE maps indicated that the amount and diversity of proteins visualized depended on the detergent that was used. Furthermore, this work revealed that the combination of detergents increased the resolution of some spots and retained the characteristics of the individual detergents, depending on their concentrations.

## Introduction

Spermatozoids are unique cells in terms of their morphology, structure, function and composition ( Rousseaux *et al*., 2005 ). They are also considered to be accessible and easily purified. Therefore, they are suitable for proteomic analysis ( Oliva *et al*., 2009 ).

Proteomic analysis using two-dimensional electrophoresis (2-DE) and mass spectrometry (MS) of sperm cells has led to a better understanding of spermatic processes, such as motility, capacitation, acrosome reaction and fertilization, and has facilitated the identification and characterization of specific spermatozoid proteins, as well as their post-translational modifications (e.g., phosphorylation, glycosylation, and methylation) ( Du Plessis *et al*., 2011 ). In addition to providing insight into the processes involved in reproduction, studies of spermatozoid proteins have allowed researchers to elucidate the causes of animal infertility ( Martínez-Heredia *et al*., 2006 ). New advances in proteomics will lead to new approaches to fertility regulation and make biotechniques such as *in vitro* fertilization viable in mammals ( Aitken and Baker 2008 ).

Many authors have described the importance of 2-DE in sperm cell proteomics studies that seek to identify the causes of infertility or to map biomarkers of fertility that can be applied to livestock. According to Yoshii *et al*., 2005 , some nucleoproteins may exhibit compositional changes, and this alteration may be a cause of human infertility. Membrane proteins are also frequently studied as they are required for the capacitation process and therefore required for fecundation ( Naaby-Habsen *et al*., 2002 ). Despite these studies, there are still challenges, which need to be addressed, that prevent the isolation of these proteins.

One challenge of sperm protein extraction is the difficulty of solubilizing certain highly hydrophobic proteins, e.g., integral plasma membrane proteins, or those possessing multiprotein complexes ( Gingras *et al*., 2007 ; Josic and Clifton, 2007 ; Tan *et al*., 2008 ; Brewis and Gadella, 2010 ). A common approach is the use of detergents that produce a hydrophilic mantle around the plasma membrane. Although this method is available, it is not very selective ( Zigo *et al*., 2011 ).

Various detergents are used in protein extraction protocols, and they act according to their physiochemical properties. Detergents destabilize cell membranes and solubilize proteins. In addition, detergents can be classified as anionic (sodium cholate and SDS), hydrophobic (Brij and Tween-20), non-ionic (Triton X-100), or zwitterionic (CHAPS), each possessing advantages and disadvantages based on their protein solubilization properties ( Maire *et al*., 2000 ; Gingras *et al*., 2007 ; Jakop *et al*., 2009 ).

An extraction protocol is considered to be ideal if it permits the solubilization of all of the proteins in a sample, eliminates contaminants, avoids protein degradation and modification, and results in good yield ( Zhen and Shi, 2011 ). Protein extraction is a crucial step in 2-DE, and the chosen extraction method must be compatible with the electrophoresis step. This study aimed to develop methodologies using CHAPS, SDS, and Triton X-100 detergents for isolating sperm proteins from Moxotó goats (* Capra hircus*), and for comparing the resulting proteomic maps.

## Materials and Methods

### Chemicals

Acrylamide, bisacrylamide, DTT, iodoacetamide, CHAPS, SDS, urea, glycerol, thiourea, TEMED, ammonium persulfate (APS), molecular markers and IPG buffer were obtained from GE Healthcare Life Sciences (São Paulo, SP, Brazil). Triton X-100, BSA and CBB were obtained from SIGMA-ALDRICH (São Paulo, SP, Brazil). Trypsin was obtained from Promega (São Paulo, SP, Brazil).

### Animals and Semen Collection

Ten Moxotó bucks from the experimental farm at the EMBRAPA Goats and Sheep Research Center in Sobral, Ceará, Brazil, were used. Semen collection was performed using an artificial vagina and a female in estrus.

### Protein Isolation

The semen samples were centrifuged at 1, 500 x g for 30 min at 5°C to separate the seminal plasma and spermatozoids. One pellet of cells corresponded to a sample pool. The spermatozoids were then washed with phosphate-buffered saline solution (PBS, pH 7.4) and centrifuged three times at 4,000 x g for 10 min at 4°C ( Novak *et al*., 2010 ). Aliquots of approximately 0.2 g of cells were separated for each extraction method. It is important to note that two sample pools were prepared from different animals: one for the first set of experiments (dataset 1) and the other for the second set (dataset 2).

The proteins were isolated using 1% or 4% CHAPS; 1% or 4% SDS; or 1% or 4% Triton X-100 (dataset 1). The CHAPS and SDS detergents were also used in the following combinations: 1% CHAPS and 1% SDS; 1% CHAPS and 4% SDS; 4% CHAPS and 1% SDS; and 4% CHAPS and 4% SDS (dataset 2). The extraction buffer consisted of detergent(s), 7 M urea, 2 M thiourea, and 20 mM DTT. A sample of 0.2 g spermatozoids was added to 300 µL of extraction buffer and stirred for two hours on ice. The samples were then centrifuged at 10,000 x g for 20 min at 4°C, and the supernatants were added to four volumes of cold 10% TCA in acetone for 16 h at 20°C as described by ( Vasconcelos *et al*., 2005 ).

### Measurement and SDS-PAGE

The proteins were quantified using the Bradford method ( Bradford, 1976 ), and protein integrity was analyzed using SDS-PAGE ( Laemmli, 1970 ).

### Two-Dimensional Electrophoresis

Para as análises proteômicas foram feitos dois géis 2D para cada tratamento. Spermatozoid proteins (250 µg) were solubilized in rehydration buffer (7 M urea, 2 M thiourea, 65 mM DTT, 1% (w/v) CHAPS, 0.5% (v/v) ampholytes, and trace amounts of bromophenol blue). The samples were applied to an IPGBox (GE Healthcare) and incubated on 7-cm immobilized pH gradient (IPG) strips with a linear pH gradient (pH 4-7) for 16 h.

Isoelectric focusing was performed using an Ettan™ IPGPhor 3™ Focusing Unit (GE Healthcare) under the following conditions: step 1, 500 V for 30 min; step 2, 4000 V for 2.5 hours; and step 3, 8000 V until reaching 18,000 total volt-hours. The strips were then stored at -80°C for later use. The strips were equilibrated in an equilibrium solution (50 mM Tris, 30% glycerol, 6 M urea, 2% SDS and trace amounts of bromophenol blue) with 1% (w/v) DTT for 15 min. The samples were then immediately incubated in an equilibrium solution containing 3% (w/v) iodoacetamide for 15 min. Finally, the proteins were separated along the second dimension using 12.5% polyacrylamide gels in the presence of SDS with 15 mA/gel for 15 min and 50 mA/gel for 4-8 hours.

### Protein staining and Analysis

Proteins were stained with CBB G-250 solution (Blue Silver) as previously described ( Candiano *et al*., 2004 ). An ImageScanner III was used to digitize the gels, and the images were managed using LabScan 6.0 software (both from GE Healthcare). The images were analyzed using ImageMaster 2D Platinum 6.0 software (GE Healthcare). The heat map and bar plot were drawn with R software using the gplots package ( http://www.r-project.org ). Pearson’s correlation co-efficient was based on the percent of spot volume in the gels.

### Mass Spectrometry

Treated spots were digested with trypsin. Digestions were performed in 50 mM ammonium bicarbonate at 1:50 w/w (enzyme/substrate). All digestions were maintained for 18 h and then stopped with 2 μL of 2% formic acid. Peptides were extracted from gel according to Shevchenko *et al*., (2006) .

The digested samples were injected using a nanoAcquity UPLC sample manager and the chromatographic separation was performed using a UPLC C18 column (75 µm x 10 cm) with a 0.35 µL/min flow rate. The mass spectra were acquired using a Synapt G1 HDMS Acquity UPLC instrument (Waters Co., Milford, MA, USA) using data-dependent acquisition (DDA) wherein the three top peaks were subjected to MS/MS. The data were processed using the Protein Lynx Global Server software (Waters Co., USA) and used for a database search using the Mascot search engine ( Perkins *et al*., 1999 ). The searches were performed by assuming a maximum of one missed trypsin cleavage, mono-isotopic peptides, partially oxidized methionine residues, and fully carbamidomethylated cysteine residues. The peptide masses and fragment mass tolerances were initially set to ± 0.1 Da for MS/MS ion searching; however, candidate peptide IDs were only accepted if the *m/z* values observed were within 0.1 Da (typically less than 0.05 Da) of the theoretical mass of the candidate ID as determined by manual review of the MASCOT search results. Os peptídeos foram identificados através de busca em banco de dados (NCBInr) utilizando ferramenta de pesquisa por padrão de fragmentação dos peptídeos nos programas ProteinLynx 2.4 (Waters Corp.) e MASCOT (Matrix Science).

## Results

### Individual Detergents

#### DE protein profile

**Figure 1:**
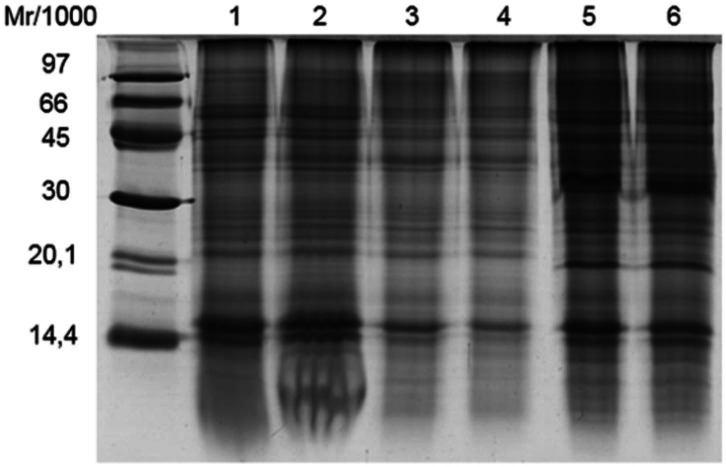
1-DE profile of *C. hircus* sperm proteins extracted using 1 - 1% CHAPS; 2 - 4% CHAPS; 3 - 1% SDS; 4 - 4% SDS; 5 - 1% Triton X-100; and 6 - 4% Triton X-100.

The 1-DE profiles obtained revealed clear bands. The overall composition of the extracted proteins appears to be consistent regardless of the extraction method; however, the results suggest that there are slight, but important, qualitative and quantitative differences. In Figure 1 , lanes 1-6 show proteins extracted using 1% and 4% CHAPS; 1% and 4% SDS; and 1% and 4% Triton X-100, respectively. Electrophoresis of proteins extracted using both concentrations of Triton X-100 revealed bands that were more intense than those produced using the other isolation methods. One of the bands was above the 30 kDa marker, and another was below 20.1 kDa.

The use of different detergent concentrations was also an important factor in the present study. Quantitative differences were found in the overall protein profiles when using either 1% SDS or 4% SDS. In contrast, no significant differences were found in the profiles of protein samples extracted using different concentrations of CHAPS or Triton X-100.

#### DE protein profiles

**Figure 2:**
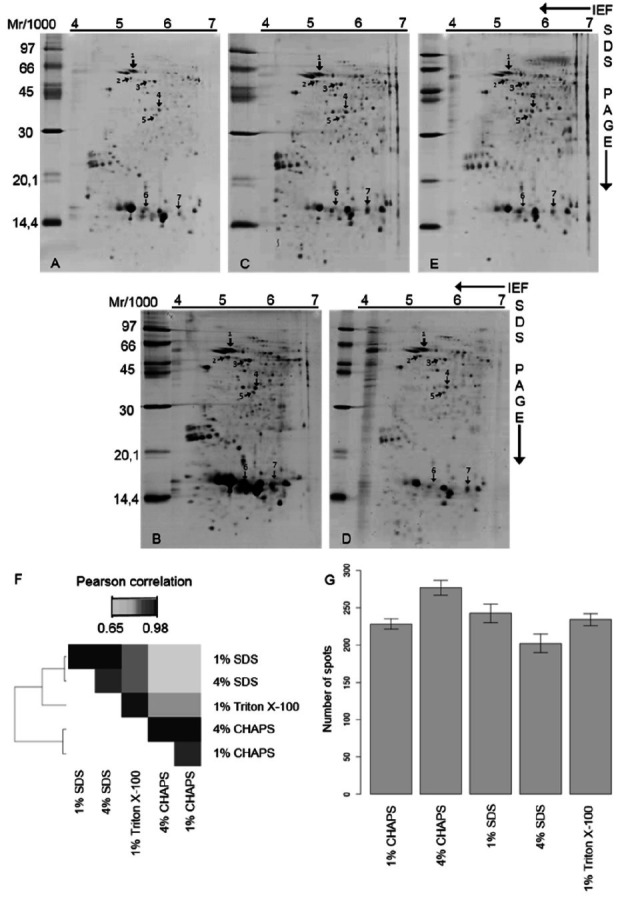
2-DE profiles of *C. hircus* sperm proteins isolated using A - 1% CHAPS (237 spots); B - 4% CHAPS (293 spots); C - 1% SDS (263 spots); D - 4% SDS (225 spots); or E - 1% Triton X-100 (248 spots). The arrows indicate quantitative and qualitative differences. F - Relationships among 2-DE protein profiles. G - Distribution of spots by gel replicates.

The 2-DE profiles were analyzed to obtain a broader view of the diversity of the proteins extracted by each detergent. Figure 2 shows the 2-D maps obtained and the relationships between them. Due to problems encountered during isoelectric focusing of samples isolated using 4% Triton X-100, the 2-D electrophoretic analysis of these samples was excluded.

The 2-DE results confirmed an overall similarity among the distribution of the extracted proteins ( Figure 2 F ); however, there seems to be a greater similarity among the SDS- and 1% Triton X-100-based isolation methods compared to the other detergents. However, upon detailed examination, the different isolation methods generated qualitatively and quantitatively distinct 2-D protein profiles. The arrows in Figure 2 indicate spots that have different intensities depending on the extraction method used, and Table 1 describes the identification of these spots. These results suggest that the detergents have different extraction efficiencies, i.e., they offer specific advantages to certain groups of proteins.

**Table 1:** Identification of proteins indicated by arrows in the 2-DE maps shown in Figure 2 .

N° Arrow	Accession	Protein name	Score	MW	pI
1	gi|676281632	Beta-1,4-galactosyltransferase 1	82	61441	7.22
2	gi|548466133	Predicted: ATP synthase subunit beta, mitochondrial	922	56148	5.14
3	gi|548515658	Predicted: Cytochrome b-c1 complex subunit 1-like	587	51307	5.84
4	gi|426249335	Predicted: Pyruvate dehydrogenase E1 subunit beta	352	39489	6.03
5	gi|28603770	F-actin-capping protein subunit beta	383	34176	6.02
6	gi|548504897	Predicted: Seminal plasma protein PDC-109-like	112	15083	5.43
7	gi|121484235	Bodhesin-2, partial	184	11885	6.75

Comparisons of the protein maps revealed that 4% CHAPS extracted the greatest diversity of proteins, followed by 1% SDS and 1% Triton X-100 ( Figure 2 G ). The results also revealed a considerable difference in the quantity of spots detected for the two CHAPS and SDS concentrations tested. This result that there are important differences in the proteins that are extracted depending on the concentration of the detergent. When 4% CHAPS was used in the extraction buffer, 17.4% more proteins were obtained than with the 1% CHAPS extraction buffer. In the case of SDS, 1% SDS extracted 16.5% more spots than 4% SDS. These results confirm that the detergent concentration is an important factor to consider when choosing an extraction protocol for proteomic analysis, as it affects both the diversity of the extracted proteins and the specific concentrations of some spots. Another important finding regarding the detergent concentration is that 1% SDS and 4% CHAPS extracted all of the proteins that were extracted by 4% SDS and 1% CHAPS. Consequently, the 17.4% and 16.5% increases observed for 1% SDS and 4% CHAPS represent relevant increases in extracted protein diversity.

#### Detergent combinations

##### DE protein profiles

**Figure 3:**
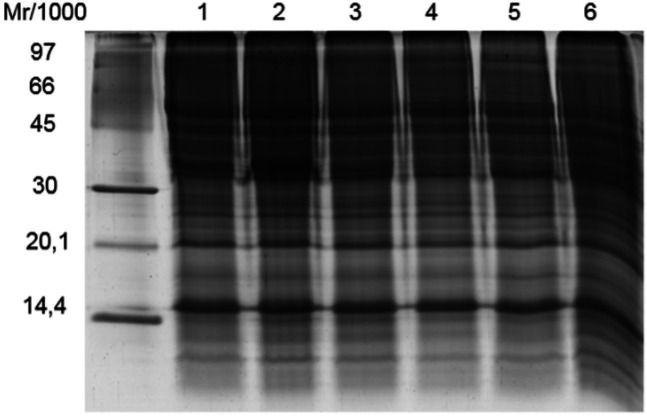
1-DE profile of *C. hircus* sperm proteins extracted using 1- 4% CHAPS; 2 - 1% SDS; 3 - 1% CHAPS and 1% SDS; 4 - 1% CHAPS and 4% SDS; 5 - 4% CHAPS and 1% SDS; and 6 - 4% CHAPS and 4% SDS.

Figure 3 shows the protein profiles obtained using the combination of CHAPS and SDS detergents. SDS-PAGE analysis revealed a profile composed of intact bands; however, the differences between the profiles obtained from combined and individual detergents were not clear by 1-DE. Consequently, 2-DE analysis was used to better visualize the diversity of the extracted proteins.

##### DE protein profiles

**Figure 4:**
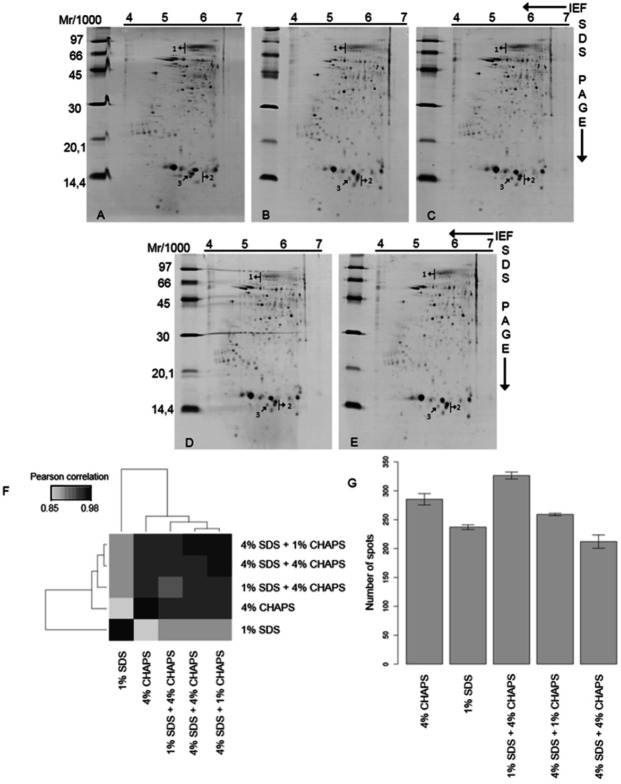
2-DE profiles of *C. hircus* sperm proteins isolated by A - 4% CHAPS (286 spots); B - 1% SDS (242 spots); C - 4% CHAPS and 1% SDS (335 spots); D - 1% CHAPS and 4% SDS (262 spots); or E - 4% CHAPS and 4% SDS (225 spots). Arrows 1, 2 and 3 indicate the regions where there was increased spot resolution, spots whose positions were modified in the presence of SDS and spots that appeared during extraction with SDS, respectively. F – Relationships among 2-DE protein profiles. G - Distribution of spots by gel replicates.

Figure 4 presents the 2-D maps of the proteins extracted by the combination of detergents. Due to problems encountered during isoelectric focusing of samples isolated by 1% CHAPS and 1% SDS, the 2-DE analyses of these samples were excluded.

Analysis of the overlap between the gels revealed that the combinations of detergents allowed for the extraction of proteins that were specific to the individual isolation conditions. This analysis also revealed increased spot resolution in certain areas ( Figure 4 - arrow 1). It is important to note that this characteristic was observed for all of the tested proportions of SDS and CHAPS; however, the highest proportion resulted from the combination of 4% CHAPS and 1% SDS, which presented an increased diversity of proteins ( Figure 4 G ). This high proportion was followed by that resulting from the combination of 1% CHAPS and 4% SDS and the combination of 4% CHAPS and 4% SDS. These latter two combinations led to a reduced number of spots compared with the 4% CHAPS and 1% SDS individual extractions, suggesting that the concentration of the combined detergents interferes with the extraction efficiency of an individual detergent.

The arrows in Figure 4 indicate specific gel regions that were obtained using detergents both individually and in combination. Arrow 1 indicates the region with increased spot resolution that seems to have resulted from the combination of detergents. In this case, 4% CHAPS extracted a larger quantity of proteins and 1% SDS resulted in better spot resolution. Arrows 2 and 3 show spots whose positions were modified or that appeared when isolated in the presence of SDS, respectively.

## Discussion

Many reports discuss the use of detergents in the extraction of sperm proteins from mice, humans, and other mammals ( Shetty *et al*., 2001 ; Josic and Clifton, 2007 ; Reese *et al*., 2010 ). Study described the use of five types of detergents for the extraction of sperm proteins from boar, including SDS, CHAPS, and Triton X-100 ( Zigo *et al*., 2011 ).

CHAPS is largely used in proteomic studies involving 2-DE and animal reproduction due to its compatibility with IEF ( Baker *et al*., 2008 ), and the same compatibility has been reported for Triton X-100 ( D’Amours *et al*., 2010 ). Another important characteristic of Triton X-100 is its exceptional efficiency in extracting detergent-resistant membrane domains ( Travis *et al*., 2001 ; Girouard *et al*., 2009 ; Jakop *et al*., 2009 ). SDS is an anionic detergent that efficiently extracts membrane proteins and protein complexes; however, a major problem with using this detergent in sperm protein isolation is its incompatibility with IEF ( Brewis and Gadella 2010 ). The three detergents used herein provided satisfactory results, and there were no significant differences in their extraction efficiencies.

Jakop *et al*. (2009) noted that the use of different detergents could lead to the release of variable quantities of proteins and lipids. The results of the dosage protein assay and 1-DE corroborate this claim. In a similar study performed by Zigo *et al*. (2011) , the authors reported qualitative and quantitative differences between the extraction methods used, consistent with the results found herein. Studies performed by Jakop *et al*. (2009) , Ignotz *et al*. (2001) , Rajeev and Reddy (2004) also reported similar results.

The work of Shetty *et al*. (2001) supported the effectiveness of certain methodologies. In their results, there were notable differences between the profiles of 2-D protein maps of human spermatozoids. Among the tested methods, only one was based on Triton X-100, at a concentration of 1%. ( Asano *et al*., 2009 ) used SDS-PAGE, and after comparing the CHAPS and Triton X-100 extractions, they observed quantitative differences for five proteins. Concentrations of 4% CHAPS and 1% Triton X-100 are widely used and have been found to be very efficient in extraction processes ( Li *et al*., 2010 ; Ijiri *et al*., 2011 ; Paasch *et al*., 2011 ).

There are few studies in the literature that specifically examine the use of 2-DE for protein extraction from sperm cells using SDS. Brewis and Gadella (2010) , in a review article, reported that SDS was the most efficient detergent for protein extraction in cases in which the proteins resisted rigorous solubilization processes. They also offered a short discussion on alternative uses of SDS, such as SDS-PAGE followed by LC-MS/MS. Regardless, 2-DE is still an important technique in studies of reproduction aimed at understanding epididymis maturation and capacitation, as well as in biotechnological tools ( Aitken and Baker 2008 ; Peddinti *et al*., 2008 ; Novak *et al*., 2010 ; Soggiu *et al*., 2013 ; Kwon *et al*., 2014 ). In this context, one can see the growing need for developing isolation methods that maximize the diversity and quantity of the extracted sperm proteins to produce more informative 2-D proteome maps. The present study will contribute to the design and evaluation of future studies involving sperm proteomes from mammals, particularly those from caprine.

One limitation of 2-DE is the sample preparation step for IEF ( López, 2007 ). In plant seed proteomics, the use of SDS for protein isolation in 2-DE is already widespread, as compatibility with IEF is possible after precipitation with acetone or acetone/TCA ( Zhen and Shi, 2011 ). Proteomic studies of sheep ( Leahya *et al*., 2011 ) and rat ( Guo *et al*., 2007 ) spermatozoids using samples obtained by SDS extraction have been successful.

In light of the results of the SDS-PAGE and 2-DE of detergent-extracted proteins, a total protein isolation procedure was performed using CHAPS and SDS in the same extraction buffer. The concentration results showed that the tested combinations of detergent concentrations resulted in good extraction yields. An isolation method similar to that used herein was also used by ( Naaby-Hansen *et al*., 1997 ) for human spermatozoid samples.

Consistent with the results of this study, ( Hochstrasser *et al*., 1988 ) found that solutions containing both SDS and CHAPS increased the 2-DE resolution. ( Nakachi *et al*., 2011 ) extracted sperm proteins from Ascidiacea using a buffer containing 4% CHAPS and 0.1% Triton X-100, and they obtained a good protein profile by 2-DE. ( Martínez-Heredia *et al*., 2008 ) also combined 1% CHAPS and 1% *n*-octyl-glucopyranoside for the extraction of human sperm proteins, and the same methodology was reported by ( Kwon *et al*., 2014 ).

Proteomic analysis depends on the use of detergents that provide the necessary quantity, quality, and diversity of proteins. The present study shows that the use of various detergents generates distinct 2-DE profiles and that changes in the concentrations of these detergents influence the results. In particular, the diversity of proteins obtained from Moxotó goat spermatozoids is affected by the choice of detergent. The extraction protocol that is chosen can determine the success or failure of the proteomic analysis. Additionally, the use of a combination of CHAPS and SDS leads to more diversity in the obtained proteins and increases the spot resolution. Thus, this combination represents an important option for protein studies; however, some spots are better extracted by individual detergents, so those detergents should be used in analyses that require the extraction of specific protein groups. It is important to note that the search for efficient and reproducible isolation methods for 2-DE remains an ongoing challenge.
